# Behçet’s Disease: A Comprehensive Overview of Symptoms, Pathology, Genetics, and Treatment

**DOI:** 10.34763/jmotherandchild.20252901.d-25-00026

**Published:** 2026-02-01

**Authors:** Arbnora Batalli, Thomas Liehr, Gazmend Temaj

**Affiliations:** Paediatric Department, University Clinical Center of Kosovo, Prishtina, Kosovo; Jena University Hospital, Friedrich Schiller University, Institute of Human Genetics, Jena, Germany; Human Genetics, College UBT, Faculty of Pharmacy Prishtina, Kosovo

**Keywords:** Rare disease, TNFα inhibitors, HLA-B*51 allele, Suggested Therapies, diagnosis of BDs, MicroRNAs in Behçet’s Disease

## Abstract

Behçet’s disease (BD, also called Behçet’s syndrome) is a complex, chronic, and multisystemic disorder characterised by recurrent oral and genital ulcers, skin lesions, and various other systemic manifestations due to underlying vasculitis. This review examines the symptoms, pathology, genetic factors, and treatment approaches associated with BD. The syndrome mainly affects populations in the Mediterranean region, the Middle East, and East Asia, with varying prevalence rates in different countries. Pathologically, BD is characterized by neutrophil infiltration and endothelial cell damage, which can lead to complications such as thrombosis and aneurysms. Genetic predisposition plays an important role — particularly via the HLA-B*51 allele — while additional non-HLA-related genetic and environmental influences further increase susceptibility to the disease. Treatment strategies have focussed on reducing inflammation and managing symptoms through a range of medications, including corticosteroids, TNFα inhibitors and emerging biologics. Recent research has highlighted the potential of microRNAs in regulating inflammatory pathways, as well as their role as biomarkers for diagnosis and treatment. Ongoing studies aim to optimize therapeutic approaches and improve treatment outcomes for this challenging disease.

## Introduction

Behçet’s disease (BD), also known as Behçet’s syndrome, is a chronic multisystemic disorder characterized by oral and genital ulcers, skin lesions, ocular lesions, changes in the gastrointestinal tract and central nervous system (CNS), and many other pathologies in the vascular, articular, cardiac, pulmonary, renal and haematological systems [1, 2]. Oral ulcers in BD occur in the buccal mucosa, on the gums, tongue and lips [3]. BD is often associated with poor oral hygiene, which manifests itself in chronic tonsillitis, periodontitis and caries [4]. The most common symptoms are the genital ulcers, which often lead to diagnoses of BD, and are therefore a good biomarker and indicator of the disease. Skin lesions are characterized by vasculitis. Thrombophlebitis is associated with blood clots as a secondary symptom of mucocutaneous BD. These symptoms occur not only on the skin, but also in other organs in the digestive, vascular, and central nervous system (5]. BD is more prevalent in the Mediterranean, Middle East, and East Asia, but is rare in Northern Europe, North America, and Australia [6] and has been reported since ancient times in the Silk Road region.

The current prevalence of BD has been reported in Turkey at 370 cases per 100,000 inhabitants, in Tunisia at 8.4 per 100,000 inhabitants(7], in Algeria and Egypt at 3.5 or 3.6 per 100,000 inhabitants [8, 9] and 17 per 100,000 in Morocco[10]. The prevalence of BD in other countries is shown in Table 1 and Table 2.

**Table 1.** Male:Female ratio and age of onset [11, 12].

**Region**	**Male: Female Ratio**	**Typical Age of Onset**
Middle East	3.4–5.3:1 (M>F)	20–40 years (mean 25–30)
Europe, Japan	F>M or near equal	20–40 years
United States	1:5 (F>M)	20–40 years

M: Male, F: Female.

**Table 2.** Prevalence of BD by Country.

**Country**	**Prevalence (case per 100,000 inhabitants)**	**Reference**
Turkey	420	[20]
Japan	7.0 – 14.6	[20]
Korea	32.8 – 35.7	[21]
China	13.5 – 20	[22]
Mongolia	2.4	[23]
Kazakhstan	10 – 15	[23]
Iran	16.7 – 80.0	[20]
Saudi Arabia	19.5	[20]
Israel	15.2	[24]
Egypt	3.6	[8]
USA	5.2	(24]
Brazil	0.3	(25]
Colombia	1.10 – 2.2	[25, 26]
Italy	3.8	[20]
Spain	5.6 – 7.5	[20]
Portugal	1.5 – 2.4	[23]
United Kingdom	14.6	[23]
Wales	11.1	[23]
France	7.1	[24]
Germany	0.6 – 1.47	[20]
Switzerland	4.03	[27]

The aim of this literature review is to provide a comprehensive and up-to-date summary of the current knowledge on Behçet’s disease (BD), focusing on its clinical spectrum, epidemiology, pathophysiology, genetic and environmental risk factors, and diagnostic criteria, as well as advances in treatment strategies. The review critically examines the underlying mechanisms of BD, including the role of vasculitis, immune dysregulation, and genetic predisposition —particularly HLA-B*51 and other susceptibility loci — while also highlighting the importance of microRNAs in the pathogenesis of the disease, and their emerging potential as biomarkers and therapeutic targets. In addition, the article evaluates the strengths and limitations of current diagnostic criteria (ISG and ICBD); summarizes findings from practice and clinical trials on established and novel pharmacologic therapies; and discusses future directions for personalized medicine in BD management.

### Pathology of Behçet’s disease

BD is a complex multisystemic disease characterised by a range of clinical symptoms that are primarily attributable to underlying vasculitis. The underlying pathological process in BD is vasculitis, which can affect both small and large blood vessels. Histological examination of the affected tissues often reveals neutrophil infiltration and endothelial cell damage, which can lead to complications, including thrombosis and aneurysm formation. Patients usually suffer from recurrent ulcers in the mouth and genital area. Biopsies show a neutrophilic inflammatory resection characterised by endothelial swelling and leukocytoclastic vasculitis. The eyes are often affected in BD; patients often suffer from uveitis, which can lead to serious complications, such as vision loss, if not treated promptly. Some patients experience neurological symptoms that can manifest as meningitis or meningoencephalitis in conjunction with vasculitis of the central nervous system. BD can also affect various other organ systems, including the gastrointestinal tract, joints, and vascular system. Although its exact aetiology is still unknown, there is evidence of dysregulated immune responses in cases of BD, including increased neutrophil activity and abnormalities in T-lymphocyte populations [13, 14].

**Figure 1.**
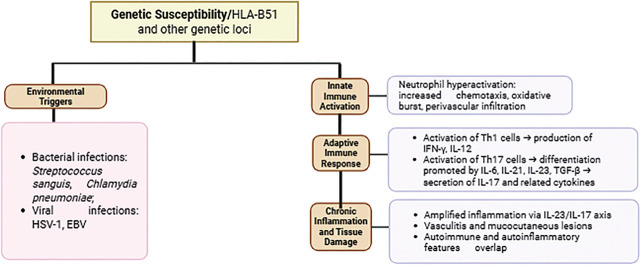
Immunopathogenesis of Behçet’s Disease.

The pathogenesis of BD centres on the hyperactivation of neutrophils, which exhibit increased chemotaxis, oxidative bursts, and perivascular infiltration, thereby contributing to tissue damage. This neutrophil hyperactivity is partly associated with the genetic risk factor HLA-B51, and is complemented by aberrant T-cell responses, particularly the activation of Th1 and Th17 subpopulations. Th1 cells produce proinflammatory cytokines such as IFN-γ and IL-12, while Th17 cells — the differentiation of which is promoted by cytokines including IL-6, IL-21, IL-23, and TGF-β — secrete IL-17 and other related cytokines that amplify inflammation and autoimmunity. The IL-23 / IL-17 axis plays a crucial role in maintaining chronic inflammation in BD [15] (Figure 1). Monocytes and macrophages also contribute to the inflammatory environment through the production of TNF-α and other cytokines [16].

### Aetiopathogenesis of Behçet’s disease

BD is highly influenced by genetic factors, with the HLA-B*51 allele being the best-known genetic risk factor for this disease. This allele is widespread in many populations, particularly in the regions along the Silk Road that historically extended from East Asia through the Middle East to the Mediterranean. Although HLA-B*51 accounts for a significant portion of genetic susceptibility to BD, its contribution to the overall genetic risk is still estimated to be less than 20% (Table 3). Other alleles, such as HLA-A*26 and HLA-Cw*1602 (Figure 1), have also been associated with the disease. Recent studies suggest that there may be additional independent genetic effects within the *HLA* region that contribute to susceptibility (Table 3).

**Table 3.** Genetic Variants of HLA-B*51 and Their Clinical Implications in Behçet’s Disease.

**Gene**	**Mutation/variant**	**Associated role in BD**	**Clinical implications**	**Reference**
HLA-B*51	Polymorphic amino acid residues (e.g., Asp at position 63, Phe at position 67)	Primary genetic risk factor for BD worldwide	Strong association with genital ulcers, ocular involvement, and skin manifestations; lower gastrointestinal involvement	[20]
HLA-B*51:01	Subtype of HLA-B*51	Most common subtype associated with Behcet’s syndrome	Linked to ocular involvement and other inflammatory symptoms	[28]
HLA-B*51:08	Subtype of HLA-B*51	Common subtype associated with Behcet’s syndrome	Also associated with ocular involvement	[28]
*ERAP1(Hap10)*	Epistatic interaction with HLA-B*51	Modifies the peptide repertoire presented by HLA-B*51, influencing immune response	Associated with altered trimming activity of MHC-Class I peptides, potentially contributing to pathogenesis	[20]

Beyond the *HLA* genes, non-*HLA* genetic factors have also been identified. These include variations in genes involved in immune regulation and inflammatory responses, such as *IL10*, *IL23R*, and *STAT4*. In addition, epigenetic influences, including microRNA polymorphisms and environmental factors, may play a role in modifying the disease’s expression and severity. A familial predisposition to BD has been observed, suggesting a hereditary component that may be more pronounced in certain ethnic groups. Overall, the genetics of BD’s pathogenesis reflect a complex interplay between multiple genetic loci and environmental factors [17,18,19].

### Suggested therapies for Behçet’s disease

Treatment for BD focuses on reducing inflammation and treating symptoms across various manifestations of the disease. The most important medications include corticosteroids, which are the mainstay for inducing remission and can be administered in oral, topical, or injection form; apremilast (Otezla), an FDA-approved selective phosphodiesterase-4 inhibitor for oral ulcers; TNFα inhibitors, such as etanercept and infliximab, which are effective for several manifestations; interferon-α, which is indicated for ocular involvement and other manifestations; and emerging biologics, such as IL-1 inhibitors, ustekinumab, and secukinumab, for refractory cases. The main goals of therapy are to relieve inflammation and symptoms, prevent serious complications associated with untreated manifestations, and to improve quality of life through symptom management [29, 30, 31] (Tables 4 and 5).

**Table 4.** Treatment Approaches for Behçet’s Disease by Manifestation.

**Manifestation**	**First-line treatments**	**Second-line treatments**	**Experimental treatments**
*Mucocutaneous*	Colchicine Glucocorticoids (low-dose oral/topical)	Azathioprine Apremilast TNFα inhibitors Interferon-α	Anti-IL-1 agents Ustekunumab Secukinumab
*Articular*	Colchicine Salazopyrin Methotrexate	TNFα inhibitors Interferon-α	Tocilizumab
*Ocular*	Azathioprine Cyclosporine A	TNFα inhibitors Interferon-α	
*Vascular*	Azathioprine (IV, oral) Cyclosporine A (IV)	TNFα inhibitors interferon-α	Tocilizumab
*Gastrointestinal*	5-Aminosalicylic acid derivatives	Glucocorticoids	Not extensively studied

**Table 5.** This table highlights recent advances in targeted therapies for Behçet’s disease, reflecting a shift toward biologics and small molecules that modulate specific immune pathways.

**Therapy**	**Study Type & Population**	**Efficacy Outcomes**	**Safety/Side Effects**	**Reference**
Infliximab	Randomized, controlled head-to-head trial vs. IFN-α in refractory BD patients	Comparable improvement in Behçet’s Disease Activity Index (BDAI) at 12 and 24 weeks; steroid-sparing effect, with 20% stopping steroids	Generally well-tolerated; trend toward better persistence and tolerability vs. IFN-α	BIO-BEHÇET’s trial [32]
Interferon-α2a	Same as above	Similar clinical efficacy to infliximab; 44% steroid cessation rate	Side effects common but manageable; slightly less tolerable than infliximab	BIO-BEHÇET’S trial [32]
Apremilast	Phase 3 paediatric oral ulcers associated with BD	Significant reduction in oral ulcer frequency and severity compared to placebo	Generally mild side effects; good safety profile in children	Ongoing Phase 3 trials[23, 33]
Filgotinib (JAK inhibitor)	Multi-centre, open-label Phase 2 trial in BD and other IMIDs	Preliminary efficacy data expected; targets JAK-STAT pathway implicated in BD inflammation	Safety data pending; JAK inhibitors have known risks, including infections and lab abnormalities	DRIMID study protocol [34]
Lenalidomide	Clinical trial in refractory mucosal BD ulcers	Evaluating efficacy and safety; lenalidomide’s immunomodulatory effects may reduce ulcer severity	Safety profile under investigation; known risks include cytopenias and thromboembolism	ClinicalTrials.gov NCT05449548
RAY121 (complement inhibitor)	Phase 1b basket trial including BD patients	Assessing safety, tolerability, and preliminary efficacy targeting classical complement pathway	Early phase; safety profile and efficacy data pending	ClinicalTrials.gov NCT06371417
Adalimumab vs Tocilizumab	Multi-centre, randomized trial in severe BD uveitis	Comparing efficacy and safety in ocular involvement; results pending	Safety profiles differ; anti-TNF and anti-IL-6R agents have distinct side effect spectra	ClinicalTrials.gov NCT05874505 [35],[36]

### The criteria used for diagnosis of BD

Due to its variable clinical presentation, the diagnosis of Behçet’s disease (BD) is based on standardized criteria.

The two most important systems are the criteria of the International Study Group (ISG) and the International Criteria for Behçet’s Disease (ICBD), which have different structures and performance metrics.

While the ISG criteria are valued for specificity in established cases, the ICBD criteria have increasingly been adopted for their sensitivity, especially in early disease or cases with neurological and / or vascular involvement [37,38,39]. Clinical assessment remains essential, especially when the criteria overlap with other diseases, such as spondyloarthropathies [39].

### Function of microRNAs in Behçet’s disease

Micro-ribonucleic acids (miRNAs) play an important role in the pathogenesis and treatment of BD. They regulate various inflammatory pathways and immune responses. The potential of miRNAs as biomarkers for diagnosis and therapeutic applications in the treatment of BD have been highlighted. Specific miRNAs are involved in the regulation and response of the various signalling pathways that play a crucial role in the manifestation of different diseases. In the case of BD, several miRNAs have been identified that can interact with signalling pathways, such as TNFs (tumour necrosis factor) [18] and IFN-γ (interferon-gamma) [40]. They have major influence on the inflammatory response process and immune process regulation. Additionally, miRNA profiles can be associated with thromboinflammation, further highlighting their role in the pathophysiology of BD. In general, miRNAs play a crucial role in the regulation of BD, but further research is needed to better understand their function and therapeutic potential [41].

The key concept for the application of miRNAs in therapy is the regulation of signalling pathways. It has been reported that miRNA-21 has the ability to control the expression of specific cytokines, such as IL-6, IL-17, and toll-like receptor 4 (TLR4). These cytokines are crucial for regulating the inflammatory mechanism in diseases such as BD [26].

Other studies have identified unique miRNAs in BD patients that are downregulated or upregulated during disease activity [41, 42].

Overall, miRNAs are good biomarkers because they support the diagnosis and monitoring of BD, with hsa-miR-224-5p, hsa-miR-206, and hsa-miR-653-5p showing diagnostic potential. They can also support distinguish between disease states and predict relapses [41, 42] (Table 7).

**Table 6.** Comparison of ISG and ICBD Diagnostic Criteria for Behçet’s Disease: Sensitivity, Specificity, and Key Limitations.

**Criteria**	**Sensitivity**	**Specificity**	**Key limitations**
ISG	66–85%	95–98%	Excludes major organ involvement; low pathergy positivity [37, 38].
ICBD	94–98%	73–97%	Risk of overdiagnosis; lower specificity [37,38,39]
The ICBD demonstrates superior accuracy (97% vs. 85% for ISG) and better accommodates early or atypical cases. However, the ISG remains widely used for its high specificity in excluding mimics.			

**Table 7.** Circulating MicroRNA Profiles Associated with Behçet’s Disease: Potential Biomarkers and Their Roles.

**miRNA**	**Description**	**Reference**
*Hsa-miR-224-5p*	Found to be significantly deregulated in patients with BS; this can potentially be used to discriminate BD from healthy controls and other diseases.	[29]
*Hsa-miR-206*	Part of the unique circulating miRNA profile associated with BD, indicating its role in the disease’s pathophysiology.	[41]
*Hsa-miR-653-5p*	Another miRNA identified in the circulating profile that may serve as a biomarker for BS diagnosis and disease activity.	[41]
*miR-21*	Associated with inflammation in BS; higher expression levels may correlate with disease activity and pathogenesis.	[43]
*miR-155*	Increased expression may indicate disease remission, and could be useful for monitoring BS progression.	[44]
*Hsa-miR-143-3p*	Identified as part of miRNA expression profiling associated with active disease states in BS.	[42]
*Hsa-miR-199a-5p*	Targeting pathways relevant to BS, this miRNA is part of the signature associated with the disease’s inflammatory response.	[42]

### Mutations of the *MVK* and *CIAS1* genes: implications for Behçet’s disease pathogenesis

**Figure 2.**
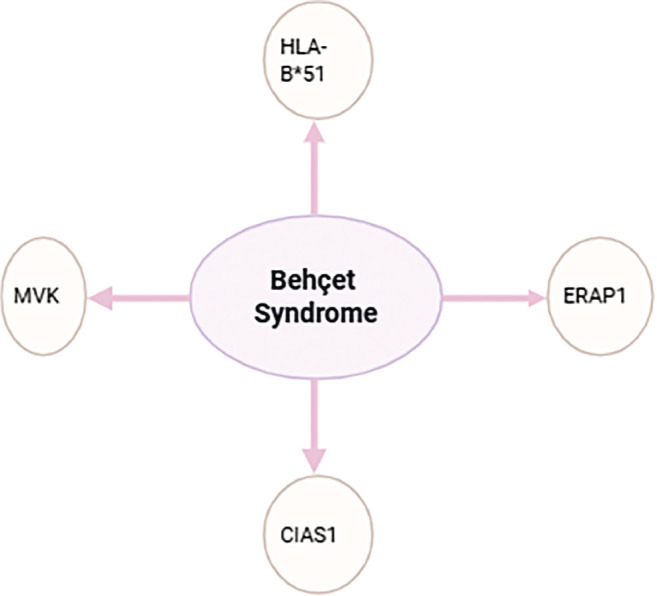
BDs association with gene mutations.

Mutations in *MVK* and *CIAS1* genes are rare, and overall are not significantly associated with BD (Figure 2).

The main functions of the *MVK* gene are central to the biosynthesis of isoprenoids. Isoprenoids are part of lipids and play various roles in cellular functions. Mutations in this gene lead to mevalonate kinase deficiency. This enzyme is responsible for the conversion of mevalonic acid into 5-phosphomevalonate. The *MVK* gene is located on the long arm of chromosome 12 and comprises 11 (10 coding) exons. Loss-of-function mutations in this gene have been associated with various diseases, including BD and mevalonate kinase deficiency (MKD). The most common mutations in MKD, among others, are loss of function / missense single nucleotide variants leading to amino acid changes; frameshifts leading to premature truncation; and intergenic deletions. This type of specific variation is involved in inhibiting the formation of a functionally active enzyme [45].

To date, more than 300 different *MVK* variants have been documented, with the p.(V377I) and p.(I268T) mutations being more common and widespread in the European population [46]. Data from the Dutch population indicate that an estimated 1 in 65 individuals carries a heterozygous pathogenic variant in the *MVK* gene. In Dutch patients diagnosed with MKD, the frequency of the p.(V377I) allele is 42% [47].

The *CIAS1* (cold-induced autoinflammatory syndrome 1) gene is located on human chromosome 1q44 and is responsible for encoding cryopyrin (also known as PYPAF1 or NALP3), a member of the pyrin gene family [48,49,50,51]. Members of this family encode various proteins that are responsible for regulating inflammatory and apoptotic processes [52]. This family, known as the pyrin protein family (also called pyrin or marenostrin), is expressed in affected tissues such as neutrophils, activated monocytes, eosinophils, synovium, and skin [53]. Patients diagnosed with FMF (Familial Mediterranean Fever) have an increased risk of developing a number of other inflammatory disorders, such as inflammatory bowel disease, vasculitis, and BD [54,55,56]. These data are very important for the risk that a mutation of pyrin may indicate and contribute to the pathogenesis of a variety of inflammatory diseases.

Mevalonate kinase-associated disorders (MKAD) are also caused by pathogenic mutations in the *MVK* gene. Boursier et al. confirmed that the prevalence is higher in patients who were compound heterozygous for p.(Ile268Thr) and p.(Val377Ile), and uncovered new associations. Patients reported as homozygous for p.(Leu264Phe), p.(Ala334Thr) or heterozygous for p.(His20Pro) and p.(Ala334Thr) were at increased risk for severe neurological or ocular symptoms. All patients homozygous for p.(Leu264Phe) had cataracts [57].

Mutations in the *MVK* and *CIAS1* genes were analysed in relation to BD (Table 8). Specific mutations in the *MVK* gene, such as V377I/V377I and V377I/S135L (c.769-38C> T and c.885+24G> A), may contribute to the genetic susceptibility to BD, especially in patients with neurological involvement (Table 5). However, other studies have not found a significantly greater number of *MVK* gene mutations in BD patients compared to control groups. *CIAS1* gene mutations have been studied only rarely; one study identified a V198M mutation in a patient with typical BD characteristics, but no symptoms of cryopyrin-associated periodic syndromes (CAPS). This suggests that *CIAS1* mutations may occur in BD, but are not a common factor. Overall, though *MVK* gene mutations may play a crucial role in the pathogenesis of BD, the results of various studies are inconsistent, and *CIAS1* gene mutations appear to be of limited relevance. Further research is needed to clarify the relationship between these two genes and BD [45, 58,59,60].

**Table 8.** Genetic Mutations Associated with Behçet’s Disease and Their Clinical Features.

**Gene**	**Mutation**	**Associated condition**	**Frequency in Behcet’s Diseases Patients**	**Clinical features**
*MVK*	V377I/V377I	Mevalonate kinase deficiency (MKD)	Found in two patients (2.06%)	Fever, chills, bipolar aphthosis, erythema nodosum, serve acne, transient arthraglia
*MVK*	V377I/S135L	Mevalonate kinase deficiency (MKD)	Found in one patient (1.03%)	Similar to above, with additional features like conjunctivitis and abdominal pain
*MVK*	V377I/-	Mevalonate kinase deficiency (MKD)	Found in one patient (1.03%)	Bipolar aphthosis, erythema nodosum, folliculitis, uveitis
*CIAS1*	V198M	Cryopyrin-associated periodic syndromes (CAPS)	Found in one patient (1.03%)	Buccal and skin aphthosis, erythema nodosum, uveitis

To date, more than 19,000 HLA class I alleles have been identified. Only four of these have reported associations with specific diseases. The most important genetic marker associated with BD is HLA-B*51 [61]. Other HLA class I alleles from this family are *HLA-B27*, which is associated with the spondyloarthritis family (ankylosing spondylitis (AS), psoriatic arthritis, reactive arthritis, and arthritis associated with inflammatory bowel disease [IBD]); *HLA-C06:02*, which is responsible for psoriasis; and *HLA-A29:02*, which is responsible for birdshot chorioretinopathy [15, 61,62,63]. Epidemiological studies show that approximately 50% to 80% of BD patients are HLA-B*51 positive, compared to approximately 20% of healthy individuals, underscoring HLA-B*51’s potential role as a risk factor for the disease. The presence of the HLA-B*51 gene in a mutated form has been shown to be associated with BD, which is associated with a higher prevalence of genital ulcers and eye involvement, while it has a negative association with gastrointestinal symptoms. The genetic interaction between the HLA-B*51 and endoplasmic reticulum aminopeptidase 1 (*ERAP1*) genes has been identified as an important factor influencing disease pathogenesis. Although HLA-B*51 is not a good indicator for the diagnosis of BD due to its occurrence in other diseases and healthy individuals, it serves as a crucial element in understanding the clinical phenotypes of this heterogeneous disease. Recent data suggest that the pathogenic mechanisms involving HLA-B*51 are not fully understood, and this underscores the need for further research to identify the specific peptides involved in disease development and their interactions with HLA-B*51. Overall, while HLA-B*51 is recognized as a marker of BD, its role extends beyond mere association to influence both susceptibility and clinical outcomes in affected individuals [20, 64, 65].

## Discussion

Behçet’s Disease (BD) is a complex multisystemic disorder characterized by a highly variable clinical spectrum, including recurrent mucocutaneous ulcers; eyesight-threatening ocular inflammation; and potentially life-threatening vascular or neurological involvement. Its global distribution, with high prevalence in the Mediterranean and Silk Road regions, as well as its marked gender and age-specific differences, reflect a complex interplay of genetic, environmental, and immunological factors in its pathogenesis. The central pathological feature of BD is vasculitis, which affects vessels of various sizes and manifests clinically from oral and genital ulcers to thrombophlebitis and aneurysms. Histopathological findings consistently show neutrophilic infiltration and endothelial damage, supporting the concept of dysregulated innate immunity as the disease’s main cause. BD’s strong association with HLA-B*51, particularly in populations along the Silk Road, remains the most robust genetic risk factor; however, this still accounts for less than 20% of genetic predisposition to the disease, with additional contributions from other HLA and non-HLA loci. Variants in genes that regulate the immune responses — such as IL10, IL23R, and STAT4 — as well as epigenetic factors, including microRNA polymorphisms, further modify susceptibility to the disease and its severity, underscoring a polygenic and multifactorial aetiology.

The diagnosis of BD is complicated by its diverse manifestations and the lack of pathognomonic laboratory markers. Although the ISG criteria offer high specificity, they may also overlook early or atypical cases. Conversely, the ICBD criteria improve sensitivity but risk overdiagnosis, especially in populations with overlapping autoimmune diseases (Table 6).

This status quo highlights the need for more objective biomarkers. A significant recent advance is the recognition that microRNAs (miRNAs) are important regulators of immune and inflammatory pathways in BD. Several miRNAs, including miR-21, miR-155, hsa-miR-224-5p, hsa-miR-206, and hsa-miR-653-5p, have been identified, the differential expression of which correlates with disease activity and corresponds to specific clinical phenotypes. These miRNAs modulate important signalling pathways, such as TNF-α and IFN-γ, and regulate cytokines, such as IL-6, IL-17, and TLR4, thereby participating in the inflammatory and vascular pathology of BD.

Circulating miRNAs are promising as minimally invasive biomarkers for diagnosis, disease monitoring, and relapse prediction. For example, hsa-miR-224-5p and hsa-miR-206 can distinguish BD from other inflammatory diseases, while miR-155 may indicate remission. However, clinical implementation of this method of diagnosis is complicated by interindividual and ethnic variability, and technical problems in standardising the assays.

The integration of genetic, epigenetic, and molecular insights, particularly with regard to miRNAs, marks a paradigm shift in BD research, with miRNA-targeted therapies representing a novel approach to modulate aberrant immune responses in refractory cases. The convergence of multi-omics technologies is likely to lead to composite biomarker panels that improve diagnostic accuracy and enable personalized treatment strategies. Nonetheless, gaps remain in the complete elucidation of the mechanistic role of miRNAs in BD pathogenesis. Large-scale, multi-ethnic validation studies are needed to confirm miRNAs’ usefulness as biomarkers or therapeutic targets. Furthermore, the complex interplay between genetic predisposition, environmental triggers, and epigenetic regulation warrants deeper investigation to advance precision medicine in BD.

## Conclusion

In summary, BD is an example of the challenges involved in diagnosing and treating a complex, multisystemic disease with variable presentation and outcome. Recent advances, particularly in the understanding of miRNAs, offer hope for more objective biomarkers and targeted therapies. In the future, interdisciplinary research integrating clinical, genetic, and molecular data will be essential to fully realising the promise of precision medicine in Behçet’s Disease.
